# Design, Synthesis and Biological Evaluation of Novel Substituted *N*,*N*′-Diaryl ureas as Potent *p*38 Inhibitors

**DOI:** 10.3390/molecules200916604

**Published:** 2015-09-11

**Authors:** Dianxi Zhu, Xingzhou Li, Wu Zhong, Dongmei Zhao

**Affiliations:** 1 Key Laboratory of Structure-Based Drug Design and Discovery of Ministry of Education, Shenyang Pharmaceutical University, Shenyang 110016, China; E-Mail: zhudianxi@126.com; 2 Laboratory of Computer-Aided Drug Design & Discovery, Beijing Institute of Pharmacology and Toxicology, Beijing 100850, China; E-Mail: xingzhou1970@gmail.com

**Keywords:** kinase inhibitor, *p*38 inhibitors, *p*38 MAPK, TNF-α, glycine flip

## Abstract

A novel series of substituted *N*,*N′*-diaryl ureas that act as *p*38α inhibitors have been designed and synthesized based on two key residues (Gly110 and Thr106) that are different in *p*38α MAPK than in other kinases. Preliminary biological evaluation indicated that most compounds possessed good *p*38α inhibitory potencies. Among these compounds, **9g** appeared to be the most powerful and is the main compound that we will study in the future.

## 1. Introduction

*p*38α mitogen-activated protein (MAP) kinase belongs to the serine/threonine family of kinases and plays an important role in regulating the production of inflammatory cytokines, including tumor necrosis factor-alpha (TNF-α) and interleukin-1b (IL-1β) [1]. In its activated state, *p*38α phosphorylates a range of intracellular protein substrates that post-transcriptionally regulate the biosynthesis of TNF-α and IL-1β. Excessive levels of TNF-α and IL-1β are thought to be responsible for the progression of many inflammatory diseases such as rheumatoid arthritis, psoriasis and inflammatory bowel disease [2,3]. To date, many research programs have focused on the inhibition of TNF-α production, antagonism of TNF-α. Therefore, *p*38α mitogen-activated protein (MAP) kinase has attracted considerable attention as a molecular target for the treatment of these conditions. Furthermore, the development of *p*38α MAP kinase inhibitors into anti-inflammatory drugs was obstructed by their severe liver toxicity and lack of kinase selectivity, as SB203580 and BIRB-796 were found to interact with the hepatic cytochrome P450 enzymes involved in drug metabolism [4,5,6,7]. Therefore searching for potent selective *p*38α inhibitors has become a research focus.

**Figure 1:**
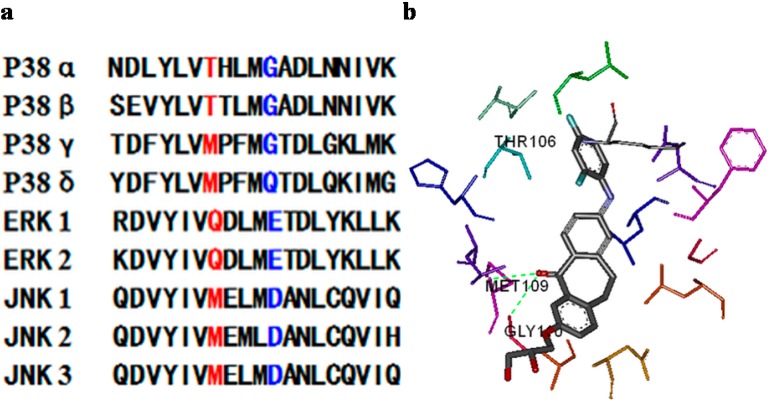
(**a**) Sequence alignment of human MAP kinases (residues 100–118) and related kinase: both Thr106 (**red**) and Gly110 (**blue**) are unique in *p*38α/β, and allow for close interactions of the proteins with the *p*38α inhibitors [13]; (**b**) Schematic of the binding mode of 2-(2,4-difluoro-phenylamino)-7-(2*R*,3-dihydroxy-propoxy)-10,11-dihydro-dibenzo[*a*,*d*]cyclohepten-5-one (Skepinone-L, PDB ID 3QUE) with *p*38α MAP kinase [14].

Members of the MAP kinase family share similar sequence and conserved structural motifs, and are all activated by dual phosphorylation of conserved threonine and tyrosine residues in the activation loop. A reasonable strategy to improve the activity and selectivity of *p*38α MAPK inhibitors is to take advantage of the key residues that are different between *p*38α MAPK and other kinases. Sequence alignment of human MAP kinases and related kinases showed that Thr106 and Gly110 are unique in *p*38α and β (Figure 1a), and these two residues are both responsible for the *p*38α selectivity of the inhibitors pyridinylimidazole, triazolopyridylbenzamides and disubstituted dibenzosuberones (Figure 1b). The high selectivity of *p*38α inhibitors has been attributed to the presence of Thr106 (a small gatekeeper) in the *p*38α ATP-binding site (residues 100–118, which cover hydrophobic region I, linker region and hydrophobic region II [8]. However, MAP kinases other than *p*38β have either a methionine or a glutamine residue in this position which prevents the binding of phenyl ring inhibitors to hydrophobic region I [9,10,11,12]. Gly110 is a residue that is specific to *p*38α, β and γ isoforms, and larger residues that are present in other MAP kinases would make the peptide chain rotation much more difficult, explaining the high activity and selectivity of most compounds that are able to form a hydrogen bond with Gly110 [8]. Therefore, the special amino acids (Gly110/Thr106) were considered as the key factor of our *p*38α inhibitor design.

**Figure 2:**
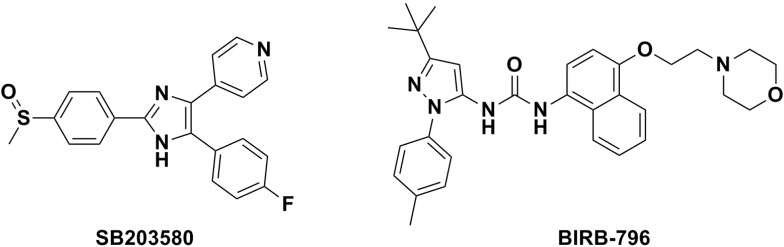
Chemical structures of SB-203580 and BIRB-796.

*p*38α inhibitors can be classified into two types based on their mode of action: ATP-competitive *p*38α inhibitors (e.g., SB203580), and non-competitive inhibitors (e.g., BIRB-796) (Figure 2). Like other kinase inhibitors, first generation *p*38α MAPK inhibitors like the pyridinyl-imidazole (SB203580) target the ATP binding site of the kinase in its active conformation. Type II inhibitors typically use the ATP binding site, but they also exploit unique hydrogen bonding and hydrophobic interactions made possible by the DFG residues of the activation loop being folded away from the conformation required for ATP phosphate transfer [15]. The *N*,*N*′-diaryl urea compounds (BIRB796) inhibit *p*38α by stabilizing a conformation of the kinase that is incompatible with ATP binding [16]. The X-ray co-crystal structure of human *p*38α MAP kinase and BIRB-796 shows that conserved residues Asp168-Phe169-Gly170 (DFG) move to a new position during binding [17,18,19,20]. In the new conformation (DFG-out), ATP binding to *p*38α MAP kinase is inhibited. The non-competitive inhibitors target the unique DFG-out inactive kinase conformation, they are likely to possess greater cellular potency and altered selectivity relative to their ATP-competitive counterparts. Ususlly tape II inhibitors have a better kinase selectivity, because the allosteric site could provide another handle for tuning kinase selectivity [15,21]. Therefore, in this context, we focused on the discovery of novel non-competitive *p*38α inhibitors using BIRB-796 as a lead that the compounds could form a hydrogen bond with Gly110. In BIRB-796, the ethoxy morpholine group forms hydrogen bond interactions with the residue of Met109, for our compounds, we attempted to replace the ethoxy morpholine group with much more rigid benzo [*d*] thiazol-2-amine group so as to form a hydrogen bond with Gly110. Furthermore, we replaced the naphthalene group of BIRB-796 with a benzyl or 5-fluorobenzyl group, which generated synthetic flexibility for structural modification of these compounds and maintained connection with the small gatekeeper. We hypothesized that structural modification of the inhibitors based on the key residues that are different between *p*38α MAPK and related kinases would generate highly potent *p*38α inhibitors.

## 2. Results and Discussion

**Scheme 1:**
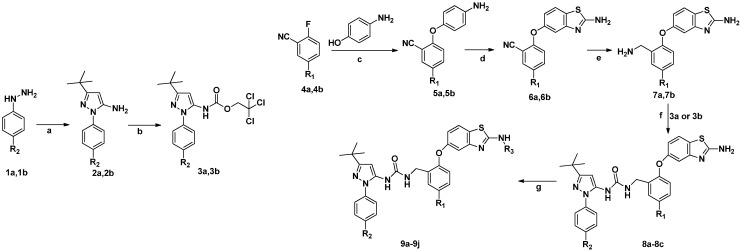
Synthesis of substituted *N*,*N*′-diaryl ureas derivatives.

Based on the above hypothesis, a novel series of *N*,*N*′-diaryl urea *p*38α inhibitors were designed and synthesized, with the aim of developing compounds with tight interactions with residues Thr106 and Gly110. The synthesis of target compounds **8a**–**8c** and **9a**–**9j** is outlined in Scheme 1. The key intermediates **7a**–7**b** were prepared from substituted 2-fluorobenzonitrile **4a**–**4b**. Treating **4a**–**4b** with 4-aminophenol generated the intermediates **5a**–**5b**. Compounds **5a**–**5b** were cyclized with KSCN and Br_2_ in the presence of acid to generate thiazoles **6a**–**6b** [22]. Intermediate **7a**–**7b** were synthesized by reduction with LiAlH_4_ in THF at room temperature or by catalytic hydrogenations under the catalysis of Pd/C [23]. For the pyrazole-benzyl moieties, substituted phenylhydrazines **1a**–**1b** were cyclized with pivaloylacetonitrile in the presence of diluted hydrochloric acid to provide compounds **2a**–**2b** [24], and **2** was treated with 2,2,2-trichloroethyl carbonochloridate to generate the intermediates **3a**–**3b**. Urea formation was achieved by coupling the corresponding benzyl amine **7a**–**7b** with carbamate **3a**–**3b** [25], to generate urea compounds **8a**–**8c** [26]. The target compounds **9a**–**9j** were synthesized by substitution of 8 with isocyanate or methyl carbonochloridate in dichloromethane at room temperature [27].

*Reactions and conditions:* (**a**) ethanol, pivaloylacetonitrile, HCl, 80 °C, 6–8 h, 86.4%; (**b**) THF, 2,2,2-trichloroethyl carbonochloridate, NaHCO_3_, 0 °C, 12 h, 85.3%; (**c**) DMSO, 4-aminophenol, K_2_CO_3_, 90 °C, 4 h, 71.1%; (**d**) AcOH, KSCN, Br_2_, 0–10 °C, 8 h, 62.5%; (**e**) THF, LiAlH_4_, 65 °C, 30–40 min, 73.7%; (**f**) DMSO, DIEA, 85 °C, 1 h, 53.5%; (**g**) DCM, RT, 1–2 h, 88.7%.

Our compounds were profiled for their *p*38α inhibitory activities and ability to inhibit TNF-α release in lipopolysaccharide (LPS)-stimulated human peripheral blood mononuclear cells (PBMCs) [28,29]. The results are presented in Table 1.Then the *p*38α kinase selectivity assay was continued and the results are presented in Table 2. Nearly all our compounds showed good activities, and good kinase selectivity with *p*38α/β showed in Table 2 which indicating that this strategy is successful. However, owing to the limited number of compounds, our knowledge of the structure-activity relationship is limited. Overall, R_1_ and R_2_ substituents have little impact on the enzyme’s activity, but TNF-α inhibition is reduced when R_2_ is a nitro group compared to when R_2_ is a different group, as in compounds **9a**, **9c**, **8a** and **8b**. The polarity of compounds with a nitro group at the R_2_ position is higher, but transmittance through the cell membrane may be lower than for compounds with a methyl group at the R_2_ position; therefore, the TNF-α inhibition activities of **8a** and **9a** in PBMC cells were lower than those of the other compounds. Secondly, the compounds with little substituents in the R_3_ position that also have the ability to form hydrogen bonds showed good activities (compounds **8a**, **8b**, **9d** and **9g**). Hydrogen bonding of the compounds with *p*38α may have been impacted by large groups at the R_3_ position (**9h**, **9i**). Finally, the activities of compounds **9h**, **9i**, and **9j** were lower than those of **9f**, **9b** and **9a**. This is probably because compounds **9h**, **9i**, and **9j** contained a fluorine atom, which may affect their ability to interact with the small gatekeeper of the *p*38α protein. However, the fluorine atom at the R_1_ position may increase kinase selectivity with *p*38α/β (**9d**, **9g**), and with a fluorine atom at the R_1_ position may allow the compounds to have lower hepatotoxicity, as the benzene ring in this position has been reported to be metabolized by CYP and biotransformed into ring epoxides, which are highly toxic metabolites [30]. Although some trends were observed, to thoroughly analyze the relationship between structure and activity, more compounds of this group will be synthesized in the future.

**Table 1:** Structure and biological activity of compounds **8a**–**8b**, **9a**–**9j**.

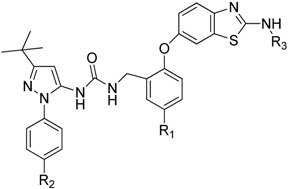

Compound No.	R_1_	R_2_	R_3_	*p*38α IC_50_ (nM) ^a^	TNF-α IC_50_ (nM) ^b^
**BIRB-796**	—	—	—	13.74 ± 2.01	n.d. ^c^
**SB203580**	—	—	—	13.89 ± 1.17	800 ± 13.15
**8a**	H	NO_2_	H	2.878 ± 0.01	31.60 ± 5.27
**8b**	F	Methyl	H	1.108 ± 0.20	8.94 ± 1.02
**9a**	H	NO_2_	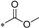	1.309 ± 0.09	33.16 ± 1.17
**9b**	H	Methyl	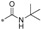	6.165 ± 0.07	62.11 ± 5.01
**9c**	H	Methyl	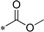	1.023 ± 0.09	18.32 ± 4.20
**9d**	H	Methyl	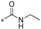	1.501 ± 0.24	8.86 ± 3.32
**9e**	H	Methyl	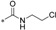	3.288 ± 0.25	90.21 ± 6.12
**9f**	H	Methyl	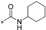	4.499 ± 0.04	56.44 ± 4.09
**9g**	F	Methyl	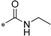	0.844 ± 1.04	6.22 ± 2.07
**9h**	F	Methyl	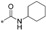	12.38 ± 0.18	995.97 ± 9.11
**9i**	F	Methyl	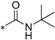	7.538 ± 1.99	106.62 ± 4.10
**9j**	F	Methyl	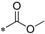	4.277 ± 1.14	52.82 ± 8.03

^a^ *p*38α MAP kinase activity was assessed based on the rate of phosphorylation of ATF-2 (activation transcription factor 2) in an *in vitro* assay; ^b^ LPS-induced TNF-α production assay in human peripheral blood mononuclear cell (PBMC); ^c^ n.d.: not determined.

**Table 2:** Kinase selectivity assay of selected compounds (**9d**, **9g**).

Compound No.	IC_50_ (nM)			
	*p*38α	*p*38β	*p*38γ	*p*38δ
**9d**	1.501 ± 0.20	17.54 ± 1.25	191 ± 7.20	230.9 ± 7.15
**9g**	2.844 ± 0.65	20.07 ± 1.40	424.3 ± 5.75	948.4 ± 8.35

**Figure 3:**
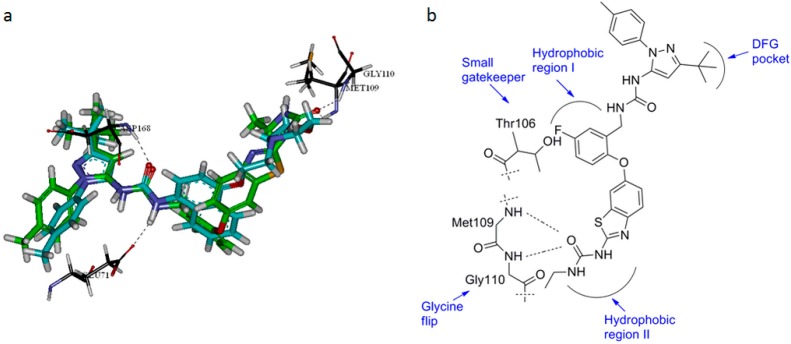
(**a**) A docking model of the *p*38α/9g complex was built on the basis of the *p*38α/BIRB796 structure (PDB code: 1KV2) [31] using the program Discovery Studio; (**b**) Binding mode of compound **9g**.

To illustrate the structure-activity relationship of **9g** and *p*38α protein, a docking model of the *p*38α/compound **9g** complex was built using Discovery studio (PDB code: 1KV2), and the results are shown in Figure 3. The docking model of **9g** with *p*38α protein reveals a hydrogen bond formed by one hydrogen of the urea group and the carboxylate oxygen of Glu71 and a hydrogen bond formed by oxygen of the urea group and N-H of Asp168. The two structures of **9g** and BIRB-796 overlap each other, and the key interactions in the *p*38α active site, consistent with previous reports, are highlighted (Figure 3a). In addition, the two primary differences (Gly110/Thr106) between *p*38α MAPK and other kinases could improve the selectivity of a *p*38α MAPK inhibitor. Firstly oxygen atoms in **9g** directly interact with the N-H of Met109 and Gly110 (Figure 3a), which can explain the high activity and selectivity of **9g** discussed previously, and perhaps compounds that bind the glycine-flipped form of *p*38α MAPK dramatically lose their potency when glycine is replaced with another amino acid, which disables the flip. Secondly, **9g** has a directly linked aromatic ring system and makes good use of the small gatekeeper (Thr106) by forming a tight complementary surface with hydrophobic region I of the enzyme (Figure 3b).

## 3. Experimental Section

### 3.1. General Synthetic Information and Synthesis Procedures

All reagents and solvents were used as received from commercial sources. ^1^H-NMR and ^13^C-NMR spectra were recorded at 400 MHz and 100 MHz on a JNM-ECA-400 instrument in CDCl_3_ or DMSO-*d*_6_, respectively. Proton and carbon chemical shifts are expressed in parts per million (ppm) relative to internal tetramethylsilane (TMS) and coupling constants (*J*) are expressed in Hertz (Hz). The splitting pattern abbreviations are as follows: multiplicity (s: singlet, d: doublet, dd: double doublet, ddd: double double doublet, dm: double multiplet, ds: double single, dt: double triplet, t: triplet, td: triple doublet, tm: triple multiplet, tt: triple triplet, q: quartet, quint: quintuplet, m: multiplet, br: broad). Low-resolution mass spectra were obtained using an API 3000 LC/MS with an ESI source or an Agilent 620B TOF LC/MS with an ESI source.

### 3.2. Chemistry

*3-(Tert-butyl)-1-(p-tolyl)-1H-pyrazol-5-amine* (**2a**). A solution of 4-tolyllhydrazine hydrochloride (5.20 g, 33 mmol) and pentylacyl acetonitrile (3.75 g, 30 mmol) in 0.4 M ethanolic solution of HCl (100 mL) was heated under reflux during 8 h. After cooling to room temperature, 1 M NaOH was added to the mixture until the pH reached 10–11. The mixture was partitioned between water and ethyl acetate. The water phase was extracted twice with dichloromethane. The organic phases were combined and washed with water and brine, then dried with Na_2_SO_4_. Evaporation of the solvent in vacuo afforded the crude product, which was subjected to recrystallization from ethyl acetate and petroleum ether to produce compound **2a** as a white solid (5.88 g,). Yield: 86.4%. ^1^H-NMR (CDCl_3_, 400 MHz), δ: 7.40 (d, 2H), 7.25 (d, 2H), 5.50 (s, 1H), 4.72 (brs, 2H), 2.37 (s, 3H), 1.32 (s, 9H). ESI-MS (+Q, *m*/*z*): 230 [M + H]^+^, 252 [M + Na]^+^*.*

*2,2,2-Trichloroethyl(3-(tert-butyl)-1-(p-tolyl)-1H-pyrazol-5-yl)carbamate* (**3a**). The **2a** (0.80 g, 3.5 mmol) was put in a 100 mL three-necked bottle and then dissolved in 30 mL THF . The bottle was cooled to 0 °C and then put 2.9 g NaHCO_3_ into the bottle while stirring. Then drip the 2,2,2-trichloroethyl carbonochloridate into the bottle, keep the temp for 30 min, then 0 °C for 12 h. The mixture was filtered and then extracted with ethyl acetate for 3 times. Dry the solution with sodium sulfate, concentrating then separate the production with column chromatograph. Yield: 85.2%. ^1^H-NMR (CDCl_3_, 400 MHz), δ: 9.10 (s, 1H), δ: 7.40 (d, 2H), 7.25 (d, 2H), 5.56 (s, 1H), 4.79 (s, 2H), 2.36 (s, 3H), 1.30 (s, 9H). ESI-MS (+Q, *m*/*z*): 404 [M + H]^+^, 426 [M + Na]^+^.

*2-(4-Aminophenoxy)-5-fluorobenzonitrile* (**5a**). The 4-aminophenol (5 g, 45.87 mmol) was put in a 250 mL three-necked bottle and then dissolved in 100 mL DMSO .The bottle was heated to 90 °C and put 19 g K_2_CO_3_ in to the bottle while stirring. Then drip the **4a** (7.76 g, 55.0 mmol) into the bottle and heated to 90 °C for 4 h. The mixture was put into 200 mL water and the water phase was extracted twice with ethyl acetate. The organic phases were combined and washed with water and brine, dried with Na_2_SO_4_ and the solvent removed under vacuum to yield the crude product, which was purified by column chromatography petroleum ether/ethyl acetate (5:1) to give compound **5a** as a yellow solid. Yield: 71.1%. ^1^H-NMR (CDCl_3_, 400 MHz), δ: 7.49 (m, 1H), 7.29 (m, 1H), 7.15 (d, 1H), 6.79 (d, 2H), 6.70 (d, 2H), 6.17 (s, 2H). ESI-MS (+Q, *m*/*z*): 229 [M + H]^+^, 251 [M + Na]^+^.

*2-((2-Aminobenzo[d]thiazol-6-yl)oxy)-5-fluorobenzonitrile* (**6a**). To a solution of compound **5a** (3 g, 13.2 mmol) in AcOH 20 mL was added KSCN (1.92 g, 19.8 mmol). The mixture was cooled to 0 °C and a solution of Br_2_ (13.2 mmol) in AcOH (6 mL) was added. The mixture was then stirred at room temperature for 8 h. The mixture was poured into water, basified with NH_4_OH (aq), and extracted with EtOAc. The organic layer was washed with water and brine and dried over Na_2_SO_4_. The compound **6a** was purified by column chromatography petroleum ether/ethyl acetate (1:1) to give a yellow solid. Yield: 62.5%. ^1^H-NMR (CDCl_3_, 400 MHz), δ: 7.88 (d, 1H), 7.67 (m, 1H), 7.50 (m, 1H), 7.30 (m, 1H), 7.18 (d, 1H), 7.15 (m, 1H), 7.01 (d, 2H). ESI-MS (+Q, *m*/*z*): 286 [M + H]^+^, 308 [M + Na]^+^.

*6-(2-(Aminomethyl)-4-fluorophenoxy)benzo[d]thiazol-2-amine* (**7a**). To 15mL of anhydrous THF was added 0.35 g (9 mmol) LiAlH_4_ in a 50 mL bottle, the bottle was kept in ice bath. Take 0.85 g (3 mmol) compound 6a and dissolve in 15 mL anhydrous THF. Drip the solution into the LiAlH_4_ suspension then stirred at 65 °C for 30–40 min. The mixture was added ethanol till no bubble generates. Then filter and concentrate the suspension. The product was separated by column chromatograph (dichloromethane/methanol 100:2), Yield: 73.7%. ^1^H-NMR (CDCl_3_, 400 MHz), δ: 8.70 (s, 1H), 7.85 (d, 1H), 7.65 (m, 1H), 7.48 (m, 1H), 7.30 (m, 1H), 7.18 (d, 1H), 7.15 (m, 1H), 7.01 (d, 2H), 4.36 (d, 1H). ESI-MS (+Q, *m*/*z*): 290 [M + H]^+^, 312 [M + Na]^+^.

*1-(2-((2-Aminobenzo[d]thiazol-6-yl)oxy)-5-fluorobenzyl)-3-(3-(tert-butyl)-1-(p-tolyl)-1H-pyrazol-5-yl)urea* (**8b**). The 6-(2-(aminomethyl)-4-fluorophenoxy)benzo[d]thiazol-2-amine **7a** (1 g, 3.5 mmol) was put in a 100 mL three-necked bottle and then dissolved in 50 mL DMSO. Then drip the 3a (1.7 g, 4.2 mmol) and 1 mL Et_3_N into the bottle and heated to 85 °C for 1 h. The mixture was put into 200 mL water and the water phase was extracted twice with ethyl acetate. The organic phases were combined and washed with water and brine, dried with Na_2_SO_4_ and the solvent removed under vacuum to yield the crude product, which was purified by column chromatography petroleum ether/ethyl acetate (5:1) to give compound **8b** as a yellow solid. Yield: 53.5%. ^1^H-NMR (DMSO, 400 MHz), δ: 8.58 (s, 1H), 8.32 (d, 2H), 7.82 (d, 2H), 7.41 (s, 2H), 7.25 (m, 3H), 7.06 (m, 2H), 6.98 (m, 1H), 6.85 (m, 1H), 6.32 (s, 1H), 4.29 (d, 2H), 1.27 (s, 9H). ESI-MS (+Q, *m*/*z*): 545 [M + H]^+^, 567 [M + Na]^+^. 145.1–147.2 °C.

*1-(3-(Tert-butyl)-1-(p-tolyl)-1H-pyrazol-5-yl)-3-(2-((2-(3-ethylureido)benzo[d]thiazol-6-yl)oxy)-5-fluorobenzyl)urea* (**9g**). The **8b** (1 g, 1.8 mmol) was put in a 100 mL three-necked bottle and then dissolved in 50 mL DCM . Then drip the ethyl isocyanate (0.4 g, 5.4 mmol) into the bottle, and the mixture was stirred at room temperature for 2 h. The mixture was put into 100 mL water and the water phase was extracted twice with DCM. The organic phases were combined and washed with water and brine, dried with Na_2_SO_4_ and the solvent removed under vacuum to yield the crude product, which was purified by column chromatography petroleum ether/ethyl acetate (3:1) to give compound **9g** as a white solid. Yield: 88.7%. ^1^H-NMR (DMSO, 400 MHz), δ: 10.64 (s, 1H), 8.28 (s, 1H), 7.48 (d, 2H), 7.35 (d, 2H), 7.31 (s, 2H), 7.25 (m, 2H), 7.06 (m, 2H), 6.98 (m, 1H), 6.85 (m, 1H), 6.22 (s, 1H), 4.29 (d, 2H), 3.16 (m, 2H), 2.49 (s, 3H), 1.27 (s, 9H), 1.06 (m, 3H). ^13^C-NMR (100 MHz, DMSO *d*_6_), δ: 160.90 (1C, thiazole), 159.89 (1C, pyrazole), 157.51 (1C, CO), 155.05 (1C, CO), 154.19 (1C, pyrazole), 153.16, 150.91, 138.02, 136.99, 136.80, 133.71, 133.35, 130.05, 124.56, 121.03, 120.74, 117.33, 115.63, 115.38, 115.06, 111.04 (18C, Ar-C), 96.22 (1C, pyrazole), 38.54 (1C, N-CH_2_), 34.80 (1C, N-CH_2_CH_3_), 32.49 (1C, CH_3_-C), 30.72 (3C, C-CH_3_), 21.09 (1C, Ar-CH_3_), 15.67 (1C, CH_2_-CH_3_). ESI-MS (+Q, *m*/*z*): 616 [M + H]^+^, 638 [M + Na]^+^. mp 197.7–199.9 °C.

*3-(Tert-butyl)-1-(4-nitrophenyl)-1H-pyrazol-5-amine* (**2b**). The title compound was obtained similarly to **2a**. ^1^H-NMR (CDCl_3_, 400 MHz), δ: 8.40 (d, 2H), 8.15 (d, 2H), 5.50 (s, 1H), 4.72 (brs, 2H), 1.32 (s, 9H). ESI-MS (+Q, *m*/*z*): 261 [M + H]^+^, 283 [M + Na]^+^.

*2,2,2-Trichloroethyl (3-(tert-butyl)-1-(4-nitrophenyl)-1H-pyrazol-5-yl)carbamate* (**3b**). The title compound was obtained similarly to **3a**. ^1^H-NMR (CDCl_3_, 400 MHz), δ: 9.10 (s, 1H), 8.40 (d, 2H), 8.15 (d, 2H), 5.56 (s, 1H), 4.79 (s, 2H), 1.30 (s, 9H). ESI-MS (+Q, *m*/*z*): 435 [M + H]^+^, 457 [M + Na]^+^.

*2-(4-Aminophenoxy)benzonitrile* (**5b**). The title compound was obtained similarly to **5a**. ^1^H-NMR (CDCl_3_, 400 MHz), δ: 7.69 (m, 1H), 7.49 (m, 1H), 7.29 (m, 1H), 7.15 (d, 1H), 6.79 (d, 2H), 6.70 (d, 2H), 6.17 (s, 2H). ESI-MS (+Q, *m*/*z*): 211 [M + H]^+^, 233 [M + Na]^+^.

*2-((2-Aminobenzo[d]thiazol-6-yl)oxy)benzonitrile* (**6b**). The title compound was obtained similarly to **6a**. ^1^H-NMR (CDCl_3_, 400 MHz), δ: 7.88 (d, 1H), 7.67 (m, 2H), 7.50 (m, 1H), 7.30 (m, 1H), 7.18 (d, 1H), 7.15 (m, 1H), 7.01 (d, 2H). ESI-MS (+Q, *m*/*z*): 268 [M + H]^+^, 290 [M + Na]^+^.

*6-(2-(Aminomethyl)phenoxy)benzo[d]thiazol-2-amine* (**7b**). The title compound was obtained similarly to **7a**. ^1^H-NMR (CDCl_3_, 400 MHz), δ: 8.70 (s, 1H), 7.85 (d, 1H), 7.65 (m, 1H), 7.48 (m, 2H), 7.28 (m, 1H), 7.18 (d, 1H), 7.15 (m, 1H), 6.98 (d, 2H), 4.34 (d, 1H). ESI-MS (+Q, *m*/*z*): 272 [M + H]^+^, 294 [M + Na]^+^.

*1-(2-((2-Aminobenzo[d]thiazol-6-yl)oxy)benzyl)-3-(3-(tert-butyl)-1-(4-nitrophenyl)-1H-pyrazol-5-yl)urea* (**8a**). The title compound was obtained similarly to **8b**. ^1^H-NMR (DMSO, 400 MHz), δ: 8.58 (s, 1H), 8.32 (d, 2H), 7.82 (d, 2H), 7.41 (s, 2H), 7.25 (m, 4H), 7.06 (m, 2H), 6.99 (m, 1H), 6.87 (m, 1H), 6.32 (s, 1H), 4.29 (d, 2H), 1.27 (s, 9H). ESI-MS (+Q, *m*/*z*): 558 [M + H]^+^, 580 [M + Na]^+^. mp 135.2–137.9 °C.

*Methyl(6-(2-((3-(3-(tert-butyl)-1-(4-nitrophenyl)-1H-pyrazol-5-yl)ureido)methyl)phenoxy)benzo[d]thiazol-2-yl)carbamate* (**9a**). The **8a** (1 g, 1.8 mmol) was put in a 100 mL three-necked bottle and then dissolved in 50 mL DCM . Then drip the methyl chloroformate (0.5 g, 5.4 mmol) into the bottle, and the mixture was stirred at room temperature for 2 h. The mixture was put into 100 mL water and the water phase was extracted twice with DCM. The organic phases were combined and washed with water and brine, dried with Na_2_SO_4_ and the solvent removed under vacuum to yield the crude product, which was purified by column chromatography petroleum ether/ethyl acetate (3:1) to give compound **9a** as a white solid. Yield: 85.6%. ^1^H-NMR (DMSO, 400 MHz), δ: 12.06 (s, 1H), 8.88 (s, 1H), 8.38 (d, 2H), 7.85 (d, 2H), 7.61 (s, 2H), 7.55 (m, 2H), 7.26 (m, 2H), 7.08 (m, 1H), 7.01 (m, 1H), 6.32 (s, 1H), 4.29 (d, 2H), 3.86 (s, 3H), 1.27 (s, 9H). ESI-MS (+Q, *m*/*z*): 616 [M + H]^+^, 638 [M + Na]^+^. mp 190.1–193.1 °C.

*1-(3-(Tert-butyl)-1-(p-tolyl)-1H-pyrazol-5-yl)-3-(2-((2-(3-(tert-butyl)ureido)benzo[d]thiazol-6-yl)oxy)benzyl)urea* (**9b**). The title compound was obtained similarly to **9g**. ^1^H-NMR (DMSO, 400 MHz), δ: 10.26 (s, 1H), 8.22 (s, 1H), 7.58 (d, 2H), 7.49 (d, 2H), 7.29 (m, 6H), 7.15 (m, 1H), 7.06 (m, 1H), 7.01 (m, 1H), 6.91 (m, 1H), 6.82 (s, 1H), 6.22 (s, 1H), 4.29 (d, 2H), 2.36 (s, 3H), 1.32 (s, 9H), 1.27 (s, 9H). ^13^C-NMR (100 MHz, DMSOe *d*_6_), δ: 160.42 (1C, thiazole), 159.06 (1C, pyrazole), 154.87 (1C, CO), 154.35 (1C, CO), 152.64 (1C, pyrazole), 152.24, 145.41, 137.74, 136.50, 136.24, 132.78, 130.27, 129.60, 129.05, 128.53, 124.22, 123.35, 120.54, 117.91, 117.41, 111.18 (18C, Ar-C), 95.05 (1C, pyrazole), 50.14 (N-C), 38.28 (1C,N-CH_2_ ), 32.00 (1C, CH_3_-C), 30.25 (3C, C-CH_3_), 28.74 (3C, C-CH_3_), 20.64 (1C, Ar-CH_3_). ESI-MS (+Q, *m*/*z*): 626 [M + H]^+^, 648 [M + Na]^+^. mp 200.1–201.2 °C.

*Methyl(6-(2-((3-(3-(tert-butyl)-1-(p-tolyl)-1H-pyrazol-5-yl)ureido)methyl)phenoxy)benzo[d]thiazol-2-yl)carbamate* (**9c**). The title compound was obtained similarly to **9a**. 1H-NMR (DMSO, 400 MHz), δ: 12.06 (s, 1H), 8.25 (s, 1H), 7.68 (d, 2H), 7.55 (d, 2H), 7.48 (s, 2H), 7.35 (m, 2H), 7.26 (m, 2H), 7.08 (m, 1H), 7.01 (m, 1H), 6.22 (s, 1H), 4.31 (d, 2H), 3.76 (s, 3H), 2.35 (s, 3H), 1.24 (s, 9H). ^13^C-NMR (100 MHz, DMSOe *d*_6_), δ: 160.85 (1C, thiazole), 159.62 (1C, pyrazole), 155.12 (1C, CO), 154.91 (1C, CO), 154.58 (1C, pyrazole), 153.28, 145.98, 138.31, 137.02, 136.67, 133.38, 130.87, 130.09, 129.53, 129.02, 124.73, 123.97, 121.71, 118.57, 118.21, 111.73 (18C, Ar-C), 95.74 (1C, pyrazole), 53.50 (1C, O-CH_3_), 38.71 (1C, N-CH_2_), 32.49 (1C, CH_3_-C), 30.70 (3C, C-CH_3_), 21.13 (1C, Ar-CH_3_). ESI-MS (+Q, *m*/*z*): 585 [M + H]^+^, 607 [M + Na]^+^. mp 183.1–185.7 °C.

*1-(3-(Tert-butyl)-1-(p-tolyl)-1H-pyrazol-5-yl)-3-(2-((2-(3-ethylureido)benzo[d]thiazol-6-yl)oxy)benzyl)urea* (**9d**). The title compound was obtained similarly to **9g**. ^1^H-NMR (DMSO, 400 MHz), δ: 10.66 (s, 1H), 8.22 (s, 1H), 7.68 (d, 2H), 7.55 (d, 2H), 7.48 (s, 2H), 7.41 (m, 2H), 7.35 (m, 1H), 7.30 (m, 1H), 7.29 (m, 1H), 7.26 (m, 1H), 7.23 (s, 1H), 6.22 (s, 1H), 4.31 (d, 2H), 3.23 (m, 2H), 2.35 (s, 3H), 1.24 (s, 9H), 0.96 (m, 3H). ESI-MS (+Q, *m*/*z*): 598 [M + H]^+^, 620 [M + Na]^+^. mp 186.5–188.1 °C.

*1-(3-(Tert-butyl)-1-(p-tolyl)-1H-pyrazol-5-yl)-3-(2-((2-(3-(2-chloroethyl)ureido)benzo[d]thiazol-6-yl)oxy)benzyl)urea* (**9e**). The title compound was obtained similarly to **9g**. ^1^H-NMR (DMSO, 400 MHz), δ: 10.86 (s, 1H), 8.26 (s, 1H), 7.58 (d, 2H), 7.51 (d, 2H), 7.45 (s, 2H), 7.41 (m, 2H), 7.35 (m, 1H), 7.30 (m, 1H), 7.29 (m, 1H), 7.26 (m, 1H), 7.23 (s, 1H), 6.22 (s, 1H), 4.31 (d, 2H), 3.73 (m, 2H), 3.63 (m, 2H), 2.35 (s, 3H), 1.24 (s, 9H). ESI-MS (+Q, *m*/*z*): 632 [M + H]^+^, 654 [M + Na]^+^. mp 192.5–195.1 °C.

*1-(3-(Tert-butyl)-1-(p-tolyl)-1H-pyrazol-5-yl)-3-(2-((2-(3-cyclohexylureido)benzo[d]thiazol-6-yl)oxy)benzyl)urea* (**9f**). The title compound was obtained similarly to **9f**. ^1^H-NMR (DMSO, 400 MHz), δ: 10.39 (s, 1H), 8.23 (s, 1H), 7.61 (d, 2H), 7.53 (d, 2H), 7.46 (s, 2H), 7.40 (m, 2H), 7.35 (m, 1H), 7.30 (m, 1H), 7.29 (m, 1H), 7.26 (m, 1H), 7.23 (s, 1H), 6.22 (s, 1H), 4.29 (d, 2H), 3.43 (m, 1H), 2.34 (s, 3H), 1.86 (m, 2H), 1.66 (m, 4H), 1.24 (s, 9H), 1.02 (m, 4H). ^13^C-NMR (100 MHz, DMSOe *d*_6_), δ: 160.44 (1C, thiazole), 159.32 (1C, pyrazole), 154.87 (1C, CO), 154.39 (1C, CO), 152.94 (1C, pyrazole), 152.30, 145.47, 137.76, 136.52, 136.27, 132.86, 130.29, 129.62, 129.03, 128.51, 124.22, 123.37, 120.56, 117.93, 117.43, 111.24 (18C, Ar-C), 95.10 (1C, pyrazole), 48.14 (1C, N-CH), 38.28 (1C, N-CH_2_), 33.42 (1C, cyclohexane), 32.63 (1C, cyclohexane) 32.02 (1C, CH_3_-C), 30.25 (3C, C-CH_3_), 25.14 (1C, cyclohexane), 24.53 (1C, cyclohexane), 24.28 (1C, cyclohexane), 20.64(1C, Ar-CH_3_). ESI-MS (+Q, *m*/*z*): 652 [M + H]^+^, 674 [M + Na]^+^. mp 171.3–173.3 °C.

*1-(3-(Tert-butyl)-1-(p-tolyl)-1H-pyrazol-5-yl)-3-(2-((2-(3-cyclohexylureido)benzo[d]thiazol-6-yl)oxy)-5-fluorobenzyl)urea* (**9h**). The title compound was obtained similarly to **9g**. ^1^H-NMR (DMSO, 400 MHz), δ: 10.38 (s, 1H), 8.23 (s, 1H), 7.61 (d, 2H), 7.53 (d, 2H), 7.46 (s, 2H), 7.40 (m, 2H), 7.35 (m, 1H), 7.30 (m, 3H), 6.22 (s, 1H), 4.29 (d, 2H), 3.53 (m, 1H), 2.34 (s, 3H), 1.88 (m, 2H), 1.68 (m, 4H), 1.24 (s, 9H), 0.91 (m, 4H). δ: 160.41 (1C, thiazole), 159.43 (1C, pyrazole), 157.11 (1C, CO), 154.55 (1C, CO), 153.79 (1C, pyrazole), 152.96, 150.41, 137.51, 136.42, 136.20, 133.23, 129.95, 129.55, 124.00, 120.52, 120.14, 116.83, 115.11, 114.78, 114.09, 110.54 (18C, Ar-C), 95.72 (1C, pyrazole), 48.13(1C, N-CH), 38.24 (1C, N-CH_2_), 33.39 (1C, cyclohexane), 32.60 (1C, cyclohexane) 32.00 (1C, CH_3_-C), 30.22 (3C, C-CH_3_), 25.11 (1C, cyclohexane), 24.50 (1C, cyclohexane), 24.25 (1C, cyclohexane), 20.61(1C, Ar-CH_3_). ESI-MS (+Q, *m*/*z*): 670 [M + H]^+^, 692 [M + Na]^+^. mp 170.2–172.3 °C.

*1-(3-(Tert-butyl)-1-(p-tolyl)-1H-pyrazol-5-yl)-3-(2-((2-(3-(tert-butyl)ureido)benzo[d]thiazol-6-yl)oxy)-5-fluorobenzyl)*urea (**9i**). The title compound was obtained similarly to **9g**. ^1^H-NMR (DMSO, 400 MHz), δ: 10.25 (s, 1H), 8.28 (s, 1H), 7.48 (d, 2H), 7.42 (d, 2H), 7.29 (m, 6H), 7.15 (m, 1H), 7.06 (m, 1H), 7.01 (m, 1H), 6.91 (m, 1H), 6.82 (s, 1H), 6.22 (s, 1H), 4.29 (d, 2H), 2.36 (s, 3H), 1.30 (s, 9H), 1.26 (s, 9H). ESI-MS (+Q, *m*/*z*): 644 [M + H]^+^, 666 [M + Na]^+^. mp 164.7–166.9 °C.

*Methyl(6-(2-((3-(3-(tert-butyl)-1-(p-tolyl)-1H-pyrazol-5-yl)ureido)methyl)-4-fluorophenoxy)benzo[d]thiazol-2-yl)carbamate* (**9j**). The title compound was obtained similarly to **9a**. ^1^H-NMR (DMSO, 400 MHz), δ: 12.05(s, 1H), 8.24 (s, 1H), 7.68 (d, 2H), 7.55 (d, 2H), 7.48 (s, 2H), 7.35 (m, 2H), 7.26 (m, 1H), 7.08 (m, 1H), 7.01 (m, 1H), 6.22 (s, 1H), 4.31 (d, 2H), 3.73 (s, 3H), 2.35 (s, 3H), 1.23 (s, 9H). ^13^C-NMR (100 MHz, DMSOe *d*_6_), δ: 160.29 (1C, thiazole), 159.12 (1C, pyrazole), 157.16 (1C, CO), 154.76 (1C, CO), 154.51 (1C, pyrazole), 153.23, 150.18, 146.42, 145.37, 141.73, 137.84, 136.68, 135.95, 132.92, 129.58, 127.39, 124.13, 121.25, 117.17, 114.88, 1110.57 (18C, Ar-C), 96.23 (1C, pyrazole), 53.02 (1C, O-CH_3_), 37.98 (1C, N-CH_2_), 32.01 (1C, CH_3_-C), 30.16 (3C, C-CH_3_), 20.64 (1C, Ar-CH_3_). ESI-MS (+Q, *m*/*z*): 603 [M + H]^+^, 625 [M + Na]^+^. mp 154.8–157.1 °C.

### 3.3. Pharmacology

#### 3.3.1. *In Vitro* Pharmacological Activity. IC_50_ Determi Nation of Inhibition of TNF-α Release from Isolated Human Peripheral Blood Mononuclear Cells (PBMCs) after LPS Stimulation

Peripheral venous blood from healthy, nonmedicated donors was collected using ethylenediaminetetraacetic acid (EDTA) as the anticoagulant. For PBMC preparation, samples of blood were diluted 1:1 with sterile phosphate buffered saline and then separated using SepMate tubes (No. 15450) with 15 mL lymphoprep (No. 07851), centrifuged at 1200× *g* for 30 min. Buffy coat cells were removed into PBS, centrifuged at 200× *g* for 10 min, and resuspended in PBMC assay buffer. A differential white cell count was performed, and PBMCs were diluted to 10,000 lymphocytes per mL in PBMC assay buffer. Test compounds were dissolved in DMSO and diluted in PBMC assay buffer to cover an appropriate concentration range. Samples of test compound solution or vehicle (20 μL) were added into 96-well tissue culture treated plates (Corning, Shanghai, China), and PBMC (160 μL) added to each well. The assay mixtures were incubated at 37 °C for 1 h in a humidified incubator containing an atmosphere of air supplemented with 5% CO_2_ before adding LPS (10 ng/mL). Plates were returned to the incubator for a further 18 h and then centrifuged before recovery of samples of supernatant. TNF-α in the samples was determined using an enzyme-linked immunosorbent assay (ELISA) (ebioscience No. 88-7346). Dose response curves were constructed from which IC_50_ values were calculated. At least *n* = 2 determinations were made from a single donor of PBMCs.

#### 3.3.2. *p*38α MAP Kinase Activity was Assessed Based on the Rate of Phosphorylation of ATF-2 (Activation Transcription Factor 2) in an *in Vitro* Assay

1× kinase reaction buffer composition: 50 mM HEPES (pH 7.5), 0.01% BRIJ-35, 10 mM MgCl_2_ and 1 mM EGTA. Titration MAPK14/*p*38α at 90 μM ATP: Prepare MAPK14/*p*38α in 1× kinase buffer with concentration at 500 ng/mL. Perform two-fold serial dilution using 1× kinase buffer from 500 ng/mL, 16 dose points. Add 5 μL of the serial diluted MAPK14/*p*38α into the 384-well plate in triplicate. Prepare 1 mL of 0.8 μM substrate GFP-ATF2 (19-96) and 180 μM ATP in 1× kinase reaction buffer. Start the reaction by adding 5 μL of the substrate GFP-ATF2 (19-96) and ATP solution (prepared at step *iv.*) into each well of the assay plate. Final starting concentration of MAPK14/*p*38α was 250 ng/mL; final GFP-ATF2 and ATP concentration was 0.4 μM and 90μM. Seal the assay plate and incubate for 1 h at room temperature (RT). Prepare 1 mL of antibody solution (20 mM EDTA and 4 nM Tb-antipATF2 (pThr71) Antibody in TR-FRET dilution buffer. Add 10 μL of antibody solution into each well of the assay plate and mix softly. Final EDTA concentration was 10 mM and final Tb-antipATF2 (pThr71) concentration was 2 nM. Seal the assay plate and incubate for 30 min at RT. Read TR-FRET signal on Envision 2104 plate reader.

Determination of inhibitor IC_50_ value: Add 2 μL/well of inhibitor in 0.5% DMSO at 5-fold the final assay concentration to the 384-well assay plate: For first cycle inhibitor screening, the final concentrations of inhibitors were 3333, 1111, 370, 123, 41, 13.7, 4.57, 1.52, 0.51, 0.17 and 0.056 nM (3 fold dilution, 11 dose points, 2 replicates for each dose). Adjust the inhibitor concentration according to the first cycle result. Add 4 μL/well MAPK14/*p*38α to each well of the 384-well assay plate. Incubate for 15 min at RT. To start the reaction, add 4 μL/well substrate GFP-ATF2 (19-96) and ATP in kinase reaction buffer. (a) MAPK14/*p*38α final concentration: 1 ng/mL; (b) Substrate final concentration: 0.4 μM; (c) ATP final concentration: 90 μM. Incubate for 1 h at RT. Add 10 μL/well of antibody solution. (a) EDTA final concentration: 10 mM; (b) Antibody final concentration: 2 nM. Incubate for 30 min at RT. Read TR-FRET signal on Envision 2104 plate reader.

Plot the resulting TR-FRET emission ratio against the concentration of inhibitor, and fit the data to a sigmoidal dose-response curve with a variable slope. Calculate the IC_50_ concentration from the curve.

#### 3.3.3. Kinase Selectivity Assay of Selected Compounds (**9d**, **9g**)

1× kinase reaction buffer composition: 50 mM HEPES (pH 7.5), 0.01% BRIJ-35, 10 mM MgCl_2_ and 1 mM EGTA. Titration *p*38β/γ/δ: Prepare *p*38β/γ/δ in 1× kinase buffer with concentration at 2000 ng/mL. Perform 3-fold serial dilution using 1×kinase buffer from 2000 ng/mL, 10 dose points. Add 5 μL of the serial diluted *p*38β/γ/δ into the 384-well plate in two replicates. Prepare 2 × substrate GFP-ATF2 (19-96) (0.6 μM/mL) and 10μMATP (*p*38 β), 6 μM ATP (*p*38γ/δ) in 1× kinase reaction buffer. Start the reaction by adding 5 μL of the substrate GFP-ATF2 (19-96) and ATP solution (prepared at step *iv.*) into each well of the assay plate. Final starting concentration of *p*38β/γ/δ was 1000 ng/mL; final GFP-ATF2 and ATP concentration was 0.3 μM and 5 μM ATP (*p*38β), 3 μM ATP (*p*38γ/δ). Sealthe assay plate and incubate for 1 h at room temperature (RT). Prepare antibody solution (20 mM EDTA and 3 nM Tb-antipATF2 (pThr71) Antibody in TR-FRET dilution buffer). Add 10 μL of antibody solution into each well of the assay plate and mix softly. Final EDTA concentration was 10 mM and final Tb-antipATF2 (pThr71) concentration was 1.5 nM. Sealthe assay plate and incubate for 30 min at RT. Read TR-FRET signal on Envision 2104 plate reader.

Determination of inhibitor IC_50_ value: Add 4μL/well *p*38β/γ/δ to each well of the 384-well assay plate. Add 2 μL/well of inhibitor in 0.5% DMSO at 5-fold the final assay concentration to the 384-well assay plate. For *p*38β, the final concentrations of inhibitors were 1111, 370, 123, 41, 13.7, 4.57, 1.52, 0.51, 0.17 and 0.056 nM (3 fold dilution, 10 dose points, 2 replicates for each dose). For *p*38γ/δ, the final concentrations of inhibitors were 10,000, 3333, 1111, 370, 123, 41, 13.7, 4.57, 1.52 and 0.51 nM (3 fold dilution, 10 dose points, 2 replicates for each dose). Incubate for 15 min at RT. To start the reaction, add 4 μL/well substrate GFP-ATF2 (19-96) and ATP in kinase reaction buffer. (a) *p*38β/γ/δ final concentration: 50 ng/mL, 140 ng/mL, 25 ng/mL; (b) Substrate final concentration: 0.3 μM; (c) ATP final concentration for *p*38β/γ/δ: 5 μM, 3 μM, 3 μM; (d) Final DMSO: 0.1%. Incubate for 1 h at RT. Add 10 μL/well of antibody solution. (a) EDTA final concentration: 10 mM; (b) Antibody final concentration: 1.5 nM. Incubate for 30 min at RT. Read TR-FRET signal on Envision 2104 plate reader.

Plot the resulting TR-FRET emission ratio against the concentration of inhibitor, and fit the data to a sigmoidal dose-response curve with a variable slope. Calculate the IC_50_ concentration from the curve.

#### 3.3.4. Docking (Discovery Studio)

Prepare Receptor: Firstly, download the PDB files (1KV2) at http://www.rcsb.org, add hydrogen atom and electric charge after clearing the water of the protein. Secondly, define a active site using BIRB-796 as a template.

Prepare Ligand: Draw the structure (**9g**) with chemdraw12.0 and minimize the molecule energy using the function (generate conformations).

Molecular Docking: Run the docking and select the best conformation (Figure 3) and display hydrogen bonds according to the docking results.

## 4. Conclusions

In summary, we have designed and synthesized a novel series of substituted *N*,*N*′-diaryl ureas compounds that are *p*38α inhibitors. A wide variety of substituents can be tolerated, and most compounds possessed good inhibitory potencies. However, owing to the limited number of compounds, we can only provide a limited description of the structure-activity relationship. Among these compounds, **8b**, **9d** and especially **9g** appeared to be the most potent ones, and will be the key compound that is studied in the future. The activity results indicated that this novel substituted *N*,*N*′-diaryl ureas compounds designed based on the two primary differences between *p*38αMAPK and other kinases could improve the activity of *p*38αMAPK inhibitors and may serve as a novel chemotype for the development of *p*38αMAPK inhibitors. Research on the *p*38 kinase biological actions of these compounds is ongoing, and results will be reported in due course.
